# Impact of tanezumab on health status, non-work activities and work productivity in adults with moderate-to-severe osteoarthritis

**DOI:** 10.1186/s12891-022-05029-x

**Published:** 2022-02-01

**Authors:** Philip G. Conaghan, Lucy Abraham, Lars Viktrup, Paul Cislo

**Affiliations:** 1 Leeds Institute of Rheumatic and Musculoskeletal Medicine, University of Leeds and NIHR Leeds Biomedical Research Centre, Leeds, UK; 2 Leeds Institute of Rheumatic and Musculoskeletal Medicine, Chapel Allerton Hospital, Leeds, LS7 4SA UK; 3 Pfizer Ltd, Tadworth, UK; 4 Eli Lilly and Company, Indianapolis, IN USA; 5 Pfizer Inc, New York, NY USA

**Keywords:** Daily activities, EQ-5D, Health status, Nerve growth factor, Osteoarthritis, Work productivity, WPAI

## Abstract

**Background:**

To evaluate the impact of tanezumab on health status, non-work activities, and work productivity in a pooled analysis of two large phase 3 osteoarthritis (OA) studies.

**Methods:**

Subcutaneous tanezumab (2.5 mg and 5 mg) was tested in double-blind, placebo-controlled, 16-week (NCT02697773) and 24-week (NCT02709486) clinical trials in patients with moderate-to-severe OA of the hip or knee. At baseline and week 16, all patients completed EQ-5D-5L and the Work Productivity and Activity Impairment-OA (WPAI-OA) activity impairment item. Those currently employed also completed WPAI-OA work time missed, impairment while working, and overall work impairment items. Between-group differences in least squares (LS) mean changes from baseline at week 16 were tested using analysis of covariance.

**Results:**

Of 1545 pooled patients, 576 were employed at baseline. Improvements in EQ-5D-5L index value at week 16 were significantly greater for the tanezumab 2.5-mg group (difference in LS means [95% confidence interval (CI), 0.03 [0.01, 0.05]; *p* = 0.0083) versus placebo. Percent improvements (95% CI) in activity impairment (− 5.92 [− 8.87, − 2.98]; *p* < 0.0001), impairment while working (− 7.34 [− 13.01, − 1.68]; *p* = 0.0112), and overall work impairment (− 7.44 [− 13.22, − 1.67]; *p* = 0.0116) at week 16 were significantly greater for the tanezumab 2.5-mg group versus placebo. Results for the tanezumab 5-mg group were generally comparable to the tanezumab 2.5-mg group, although, compared with placebo, percent improvement (95% CI) in work time missed was significantly greater for the tanezumab 5-mg group (− 3.40 [− 6.47, − 0.34]; *p* = 0.0294), but not the tanezumab 2.5-mg group (− 0.66 [− 3.63, 2.32]; *p* = 0.6637).

**Conclusions:**

These pooled analyses showed that health status, non-work activities, and work productivity were significantly improved following tanezumab administration, compared with placebo.

**Trial registration:**

ClinicalTrials.gov: NCT02697773, NCT02709486.

**Supplementary Information:**

The online version contains supplementary material available at 10.1186/s12891-022-05029-x.

## Introduction

Osteoarthritis (OA) has a detrimental impact on health-related quality of life [1, 2], especially when symptoms are severe [3]. Health status is worse than in the general population [4, 5]; daily activities can be difficult [6] due to pain, joint stiffness, and impact on physical functioning; and patients can experience work disability [7], reduced work productivity [7, 8], and risk of work loss [9, 10]. Standard pharmacologic treatment with agents such as acetaminophen, nonsteroidal anti-inflammatory drugs (NSAIDs), and tramadol/other opioids [11–13] can be inadequate or inappropriate [12].

The nerve growth factor monoclonal antibody tanezumab is being investigated for the treatment of moderate-to-severe OA pain. As part of the phase 3 OA program using subcutaneous administration, two randomized, placebo-controlled clinical trials were completed and the data reported separately [14, 15]. Combined, these studies provide a large data set to evaluate the effect of tanezumab compared with placebo on quality of life outcomes, including non-work activities and work productivity. This exploratory pooled analysis of these two phase 3 studies therefore evaluated the impact of tanezumab on health status, non-work activities, and work productivity.

## Methods

### Study details

Both phase 3 studies were randomized, double-blind, and placebo-controlled with subcutaneous administration of study treatment at 8-week intervals [14, 15]. Study 1, with primary endpoint at week 16, was a dose-titration study conducted in North America (ClinicalTrials.gov: NCT02697773. First submitted 11/02/2016) with three arms: placebo at baseline and week 8, tanezumab 2.5 mg at baseline and week 8, or tanezumab 2.5 mg at baseline and tanezumab 5 mg at week 8 [14]. Both tanezumab dose groups met all three co-primary endpoints, with significantly greater improvements than placebo at week 16 in Western Ontario and McMaster Universities Osteoarthritis Index (WOMAC*) Pain and Physical Function, and patient’s global assessment of OA (PGA-OA) [14]. Study 2, with primary endpoint at week 24, enrolled patients in Europe or Japan (NCT02709486. First submitted 26/02/2016) who received three doses of placebo, tanezumab 2.5 mg, or tanezumab 5 mg (at baseline, week 8, and week 16) [15]. The primary analysis of this study showed that tanezumab 2.5 mg resulted in significant improvements at week 24 in WOMAC Pain and Physical Function (though not PGA-OA), whereas tanezumab 5 mg was significant on all three co-primary endpoints [15].

**Fig. 1:**
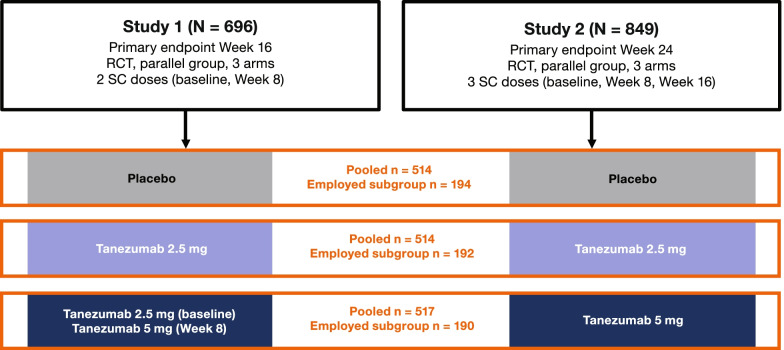
The pooling strategy. Data from Study 1 (NCT02697773) [14] dose-titration group (tanezumab 2.5 mg at baseline, tanezumab 5 mg at week 8) were pooled with the Study 2 (NCT02709486) [15] tanezumab 5 mg group for analyses at week 16. RCT, randomized controlled trial; SC, subcutaneous

Secondary efficacy data from the two studies were pooled for the current analyses at week 16, a time point common to both studies [14, 15] and the primary endpoint for the shorter of the two studies [14]. Data from the Study 1 dose-titration arm (tanezumab 2.5 mg at baseline and tanezumab 5 mg at week 8) were pooled with the Study 2 tanezumab 5 mg group (Fig. 1).

Key eligibility criteria included radiographically confirmed (Kellgren-Lawrence [KL] [16] grade ≥ 2 in the index joint) moderate-to-severe OA of the hip or knee [14, 15]. Patients were required to have WOMAC [17] Pain and Physical Function subscale scores ≥5 in the index joint and PGA-OA “fair”, “poor”, or “very poor” at baseline, and a documented history that pain relief from acetaminophen was insufficient, that pain relief from NSAIDs was inadequate or they could not be taken due to intolerance or contraindication, and that either tramadol or opioids resulted in inadequate pain relief or could not be taken due to intolerance or contraindication (or were unwilling to take opioids) [14, 15].

*© 1996 Nicholas Bellamy. WOMAC® is a registered trademark of Nicholas Bellamy (CDN, EU, USA).

### Assessments

At baseline and week 16, in both studies, all patients completed EQ-5D-5L (developed by EuroQol) [18] and the activity impairment item of the Work Productivity and Activity Impairment-OA (WPAI-OA). Those currently employed also completed WPAI-OA work time missed, impairment while working, and overall work impairment items.

The self-administered EQ-5D-5L [18] questionnaire determined current overall health status (“today”), each of five dimensions (mobility, self-care, usual activity, pain/discomfort, and anxiety/depression) being assessed on a 5-level severity scale (no/slight/moderate/severe/extreme problems). A UK value set was used to transform a health state to a single summary index value, with higher score indicating better health status. Possible scores ranged from − 0.59 (“worse than dead”) to 1.00 (the value of full health). In patients with hip or knee OA, a minimal detectable change (MDC) at the group level, which expresses the minimal magnitude of change in EQ-5D-5L between groups above which the observed change is likely to be real and not just measurement error, has been estimated to be 0.01 [19]. In addition, health status was rated on the EQ visual analog scale (VAS) in response to, “We would like to know how good or bad your health is today,” scored on a 100-mm scale (0 = the worst health you can imagine, 100 = the best health you can imagine).

The six-item, self-administered WPAI-OA of the Knee or Hip v2.0 questionnaire assessed the impact of OA over the past 7 days on four metrics [20], each subscale score being expressed as an impairment percentage (0–100%), with higher values indicating greater impairment and less productivity. Percent activity impairment was derived from the question, “During the past seven days, how much did your OA of the knee or hip affect your ability to do your regular daily activities, other than work at a job?”, which was answered on a 0 to 10 scale (0 = no effect on my daily activities, 10 = completely prevented me from doing my daily activities) and the score multiplied by 10. Those who selected “yes” to the question, “Are you currently employed?” also completed the work-related items. Percent work time missed was calculated (number of hours missed/[number of hours missed + number of hours worked] × 100) in response to the questions, “During the past seven days, how many hours did you miss from work due to problems associated with your OA of the knee or hip?” and, “During the past seven days, how many hours did you actually work?” Percent impairment while working was derived from the question, “During the past seven days, how much did your OA of the knee or hip affect productivity while you were working?”, which was answered on a 0 to 10 scale (0 = no effect on my work, 10 = completely prevented me from working) and the score multiplied by 10. Percent overall work impairment was calculated by combining absenteeism and presenteeism (% of work missed + [% of work not missed] x [% impairment while at work]). MDCs have not been published for WPAI-OA. In patients with psoriatic arthritis, individual improvements of 15–20% in WPAI items were reported to be minimal clinically important differences [21]. In Crohn’s disease, improvements of 8.5% (activity impairment), 6.5% (absenteeism), 6.1% (presenteeism), and 7.3% (overall work impairment) were reported to be minimally important differences between treatment groups [22].

### Statistical analysis

All randomized patients who received at least one dose of study medication were included in the analyses. Between-group differences in least squares (LS) mean changes from baseline at week 16 were tested using analysis of covariance (ANCOVA). No correction for multiplicity was made for these exploratory pooled analyses, and missing data were assumed to be missing at random.

SAS software version 9.4 (Cary, North Carolina, USA) was used for all statistical analyses, and *p* ≤ 0.05 was considered significant.

## Results

### Demographics and baseline characteristics

The overall population comprised a total of 1545 patients of whom 576 were employed at baseline (Table 1). There were no notable differences between the employed subgroup and the overall population, except the employed subgroup was younger and included a higher proportion of patients with hip index joints (Table 1).

**Table 1:** Demographics and baseline characteristics of the pooled population

	Overall population^**a**^			Subgroup who were employed (at baseline^**b**^)		
	Placebo (***n*** = 514)	Tanezumab 2.5 mg (***n*** = 514)	Tanezumab 5 mg (***n*** = 517)	Placebo (***n*** = 194)	Tanezumab 2.5 mg (***n*** = 192)	Tanezumab 5 mg (***n*** = 190)
Male, *n* (%)	161 (31.3)	171 (33.3)	173 (33.5)	73 (37.6)	85 (44.3)	64 (33.7)
Female, *n* (%)	353 (68.7)	343 (66.7)	344 (66.5)	121 (62.4)	107 (55.7)	126 (66.3)
Age, years, mean (SD)	62.5 (9.8)	63.2 (9.4)	63.4 (9.9)	56.3 (8.2)	57.7 (8.1)	57.4 (8.8)
White/Black or African American/Asian/other or unknown, *n*/*n*/*n*/*n*	403/60/47/4	423/43/43/5	418/50/42/7	146/24/22/2	148/22/20/2	140/22/26/2
Disease duration, years, mean (SD)^c^	8.7 (8.1)	7.9 (7.8)	8.3 (7.2)	7.5 (7.8)	7.4 (7.9)	7.5 (7.3)
Index joint, *n* (%)						
Hip	80 (15.6)	83 (16.1)	83 (16.1)	39 (20.1)	38 (19.8)	38 (20.0)
Knee	434 (84.4)	431 (83.9)	434 (83.9)	155 (79.9)	154 (80.2)	152 (80.0)
Kellgren-Lawrence grade of index joint, *n* (%)^d^						
0	0	2 (0.4)	0	–	–	–
1	0	1 (0.2)	0	0	1 (0.5)	0
2	124 (24.1)	109 (21.2)	117 (22.7)	46 (23.7)	50 (26.0)	33 (17.5)
3	221 (43.0)	232 (45.1)	226 (43.8)	94 (48.5)	78 (40.6)	94 (49.7)
4	169 (32.9)	170 (33.1)	173 (33.5)	54 (27.8)	63 (32.8)	62 (32.8)
Average pain in the index joint (pain diary) score, mean (SD)^e^	7.01 (1.48)	6.97 (1.50)	7.00 (1.46)	6.99 (1.53)	6.99 (1.40)	7.10 (1.47)
WOMAC Pain score, mean (SD)^f^	6.9 (1.1)	6.9 (1.1)	6.9 (1.1)	7.0 (1.2)	6.9 (1.1)	7.1 (1.2)
WOMAC Physical Function score, mean (SD)^f^	7.0 (1.1)	7.0 (1.0)	7.0 (1.1)	7.0 (1.2)	7.0 (1.1)	7.2 (1.1)
PGA-OA score, mean (SD)^f^	3.5 (0.6)	3.5 (0.6)	3.5 (0.6)	3.5 (0.6)	3.5 (0.6)	3.5 (0.6)
Employment status, *n* (%)						
Employed	194 (37.7)	192 (37.4)	190 (36.8)	194 (100.0)	192 (100.0)	190 (100.0)
Not employed	315 (61.3)	317 (61.7)	326 (63.1)	–	–	–
Not known/data missing	5 (1.0)	5 (1.0)	1 (0.2)	–	–	–
EQ-5D-5L, mean (SD)^f^	0.48 (0.20)	0.48 (0.19)	0.47 (0.20)	0.47 (0.19)	0.49 (0.19)	0.46 (0.21)
EQ VAS, mean (SD)^f^	60.81 (19.26)	60.21 (20.13)	59.32 (18.83)	–	–	–
Percent activity impairment, mean (SD), *n*	67.88 (14.00), 509	67.94 (15.53), 509	68.53 (14.59), 516	65.72 (15.29), 194	63.80 (18.66), 192	66.58 (16.11), 190
Percent work time missed, mean (SD), *n*	–	–	–	7.05 (18.85), 169	6.64 (17.79), 176	7.75 (19.55), 166
Percent impairment while working, mean (SD), *n*	–	–	–	58.86 (20.90), 166	59.25 (21.61), 174	58.95 (20.17), 162
Percent overall work impairment, mean (SD), *n*	–	–	–	60.88 (20.84), 166	61.07 (21.78), 174	60.41 (20.51), 162

^a^Some of these data were published previously [23]: Adapted from Schnitzer TJ, Berenbaum F, Conaghan PG, Dworkin RH, Gatti D, Yang R, et al. Single and composite endpoints of within-patient improvement in symptoms: pooled tanezumab data in patients with osteoarthritis. Rheumatol Ther. 2021;8:1759–74 (http://creativecommons.org/licenses/bync/4.0/)

^b^The number of patients employed at week 16 was *n* = 181 (placebo), *n* = 177 (tanezumab 2.5 mg), *n* = 176 (tanezumab 5 mg)

^c^Sample size *n* = 514 (placebo), *n* = 512 (tanezumab 2.5 mg), *n* = 515 (tanezumab 5 mg) for overall population, and *n* = 194 (placebo), *n* = 190 (tanezumab 2.5 mg), *n* = 189 (tanezumab 5 mg) for employed subgroup

^d^Sample size *n* = 514 (placebo), *n* = 514 (tanezumab 2.5 mg), *n* = 516 (tanezumab 5 mg) for overall population, and *n* = 194 (placebo), *n* = 192 (tanezumab 2.5 mg), *n* = 189 (tanezumab 5 mg) for employed subgroup

^e^Sample size *n* = 506 (placebo), *n* = 508 (tanezumab 2.5 mg), *n* = 511 (tanezumab 5 mg) for overall population, and *n* = 192 (placebo), *n* = 190 (tanezumab 2.5 mg), *n* = 188 (tanezumab 5 mg) for employed subgroup

^f^Sample size *n* = 513 (placebo), *n* = 513 (tanezumab 2.5 mg), *n* = 517 (tanezumab 5 mg) for overall population

*PGA-OA* Patient’s global assessment of osteoarthritis, *SD* Standard deviation, *VAS* Visual analog scale, *WOMAC* Western Ontario and McMaster Universities Osteoarthritis Index

Of the overall population, 696 were enrolled in North America [14], 743 were enrolled in Europe [24], and 106 were enrolled in Japan [24]. Across the three treatment groups (placebo, tanezumab 2.5 mg, tanezumab 5 mg) in the overall population, the index joint was a knee for 83.9–84.4% of patients, KL grade 3 for 43.0–45.1%, and KL grade 4 for 32.9–33.5% of patients, and WOMAC Pain score (mean) was 6.9 (Table 1).

At baseline across the three treatment groups (means), EQ-5D-5L index value was 0.47–0.48 and activity impairment was 67.88–68.53% in the overall population. At baseline in the employed subgroup, work time missed due to OA was 6.64–7.75%, impairment while working was 58.86–59.25%, and overall work impairment was 60.41–61.07% across the three treatment groups (Table 1).

### Health status

**Fig. 2:**
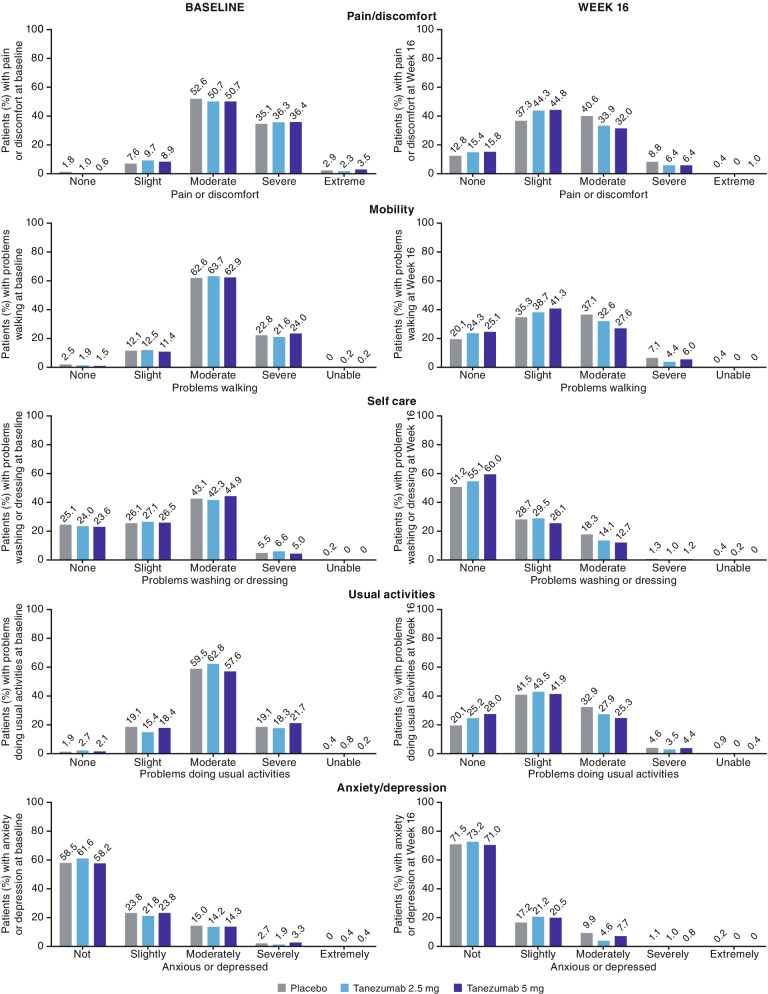
EQ-5D-5L responses at baseline and week 16. Observed data. All patients completed the EQ-5D-5L. Statistical analysis of dimension responses was not conducted. Sample sizes at baseline: *n* = 513 (placebo), *n* = 513 (tanezumab 2.5 mg), *n* = 517 (tanezumab 5 mg). Sample sizes at week 16: *n* = 453 (placebo), *n* = 481 (tanezumab 2.5 mg), *n* = 482 (tanezumab 5 mg)

Improvements were seen in all three treatment groups across the five dimensions of the EQ-5D-5L, with notably more patients in the least impaired categories and fewer patients in the most impaired categories at week 16, compared with baseline (Fig. 2).

At week 16, improvements from baseline in EQ-5D-5L index value were significantly greater for the tanezumab 2.5 mg group (LS mean difference 0.03; *p* = 0.0083) and the tanezumab 5 mg group (LS mean difference 0.04; *p* = 0.0015), compared with placebo (Table 2).

**Table 2:** Change from baseline at week 16 in health status

	Placebo (***n*** = 514)	Tanezumab 2.5 mg (***n*** = 514)	Tanezumab 5 mg (***n*** = 517)
EQ-5D-5L index value			
*n*	452	480	482
LS mean (SE) change from baseline	0.15 (0.01)	0.18 (0.01)	0.19 (0.01)
Difference in LS means (95% CI)		0.03 (0.01, 0.05)	0.04 (0.01, 0.06)
*p* value		0.0083	0.0015
EQ VAS			
*n*	452	480	482
LS mean (SE) change from baseline	10.09 (0.84)	11.72 (0.83)	12.58 (0.82)
Difference in LS means (95% CI)		1.63 (−0.40, 3.65)	2.49 (0.47, 4.52)
*p* value		0.1148	0.0157

Observed data. All patients completed the EQ-5D-5L and EQ VAS. UK value set was used. ANCOVA model with independent variables for Study 1 and Study 2: index joint stratification factor, baseline response to question, baseline diary average pain score, and treatment

*ANCOVA* Analysis of covariance, *CI* Confidence interval, *LS* Least squares, *SE* Standard error, *VAS* Visual analog scale

At week 16, improvements from baseline in EQ VAS assessment of current health status were significantly greater for the tanezumab 5 mg group (LS mean difference 2.49; *p* = 0.0157) but not the tanezumab 2.5 mg group (LS mean difference 1.63; *p* = 0.1148), compared with placebo (Table 2).

### Non-work activities and work productivity

At week 16, percent improvements from baseline in activity impairment were significantly greater for the tanezumab 2.5 mg group (LS mean difference − 5.92; *p* < 0.0001) and the tanezumab 5 mg group (LS mean difference − 5.96; *p* < 0.0001), compared with placebo (Table 3).

**Table 3:** Change from baseline at week 16 in non-work activities and work productivity

	Placebo (***n*** = 514)	Tanezumab 2.5 mg (***n*** = 514)	Tanezumab 5 mg (***n*** = 517)
Non-work activities			
Percent activity impairment			
*n*	448	476	482
LS mean (SE) change from baseline	−23.49 (1.22)	− 29.41 (1.20)	− 29.45 (1.19)
Difference in LS means (95% CI)		−5.92 (−8.87, − 2.98)	−5.96 (− 8.89, − 3.02)
*p* value		< 0.0001	< 0.0001
Work productivity			
Percent work time missed			
*n*	127	142	126
LS mean (SE) change from baseline	−0.20 (1.19)	−0.86 (1.16)	− 3.60 (1.21)
Difference in LS means (95% CI)		−0.66 (−3.63, 2.32)	− 3.40 (− 6.47, − 0.34)
*p* value		0.6637	0.0294
Percent impairment while working			
*n*	124	140	125
LS mean (SE) change from baseline	−18.59 (2.29)	−25.94 (2.22)	−26.46 (2.31)
Difference in LS means (95% CI)		−7.34 (−13.01, − 1.68)	−7.87 (− 13.71, − 2.03)
*p* value		0.0112	0.0084
Percent overall work impairment			
*n*	124	140	125
LS mean (SE) change from baseline	−19.12 (2.33)	−26.56 (2.26)	−27.49 (2.36)
Difference in LS means (95% CI)		−7.44 (−13.22, − 1.67)	−8.37 (−14.32, − 2.42)
*p* value		0.0116	0.0060

Observed data, WPAI-OA of the Knee or Hip v2.0 questionnaire. All patients completed the activity impairment item. Those currently employed also completed work time missed, impairment while working and overall work impairment items. ANCOVA model included the following independent variables for Study 1 vs Study 2, index joint stratification factor, baseline response to question, baseline diary average pain score, and treatment

*ANCOVA* Analysis of covariance, *CI* Confidence interval, *LS* Least squares, *SE* Standard error, *WPAI-OA* Work Productivity and Activity Impairment-osteoarthritis

In the employed subgroup, the percent improvement from baseline in work time missed was significantly greater for the tanezumab 5 mg group (LS mean difference − 3.40; *p* = 0.0294) but not the tanezumab 2.5 mg group (LS mean difference − 0.66; *p* = 0.6637), compared with placebo at week 16 (Table 3). The percent improvement from baseline in impairment while working was significantly greater for the tanezumab 2.5 mg group (LS mean difference − 7.34; *p* = 0.0112) and the tanezumab 5 mg group (LS mean difference − 7.87; *p* = 0.0084), compared with placebo (Table 3). The percent improvement from baseline in overall work impairment was significantly greater for the tanezumab 2.5 mg group (LS mean difference − 7.44; *p* = 0.0116) and the tanezumab 5 mg group (LS mean difference − 8.37; *p* = 0.0060), compared with placebo (Table 3).

## Discussion

These analyses of pooled data showed that patients with moderate-to-severe OA experienced greater improvement in health status, non-work activities, and work productivity at week 16 following subcutaneous tanezumab administration, compared with placebo.

The baseline health status of the current pooled population (EQ-5D-5L index value, mean 0.47–0.48; Table 1) was similar to that of patients with physician-diagnosed knee or hip OA (EQ-5D-5L index value, mean 0.532) [25] and patients with self-reported physician-diagnosed moderate or severe OA of various joints taking prescription medication (EQ-5D-5L index value, mean ~ 0.4) [3]. The health status of these populations with OA is lower than that reported for the general population (EQ-5D-5L index value, mean 0.856–0.924) [25–27]. Comparisons with other diseases (e.g. cancer, diabetes, and heart disease [27]) are confounded by methodological differences (e.g. patient inclusion criteria, disease severity). The majority of the current pooled population had moderate or severe problems with mobility and usual activities at baseline, and almost half had moderate or severe problems with self-care (Fig. 2). A large placebo response was observed across EQ-5D-5L dimensions (Fig. 2), likely reflecting the placebo effects observed on measures of pain and function in the individual studies [14, 15].

Improvements in health status were significantly greater for tanezumab than placebo in the current analyses, and the LS mean group differences relative to placebo in EQ-5D-5L index value (0.03–0.04; Table 2) were well above the published group level MDC (0.01) [19]. Changes in EQ VAS reflected those of the EQ-5D-5L index value, but did not reach significance compared with placebo for the tanezumab 2.5 mg group. Few prospective intervention studies have reported the impact of pharmacologic treatment compared with placebo on EQ-5D in patients with OA [28, 29]. Studies of tapentadol and oxycodone have reported inconsistent benefits on EQ-5D index value [30–32], and there was no improvement from baseline in EQ VAS following a single injection of hyaluronic acid for OA [33].

The impact of OA on activities of daily living is considerable [6, 34]. In the current pooled population with OA of the hip or knee, baseline activity impairment (67.88–68.53%; Table 1) was similar to that reported for patients with moderate or severe OA of various joints taking prescription medication in a cross sectional study of patients in Europe (~ 68%) [3]. The percent improvements in activity impairment were significantly greater following tanezumab treatment than with placebo in the current analyses, with LS mean improvements from baseline in all three groups (23.49–29.45; Table 3) exceeding the minimal clinically important difference (individual patient change) of 20% reported for psoriatic arthritis [21], although the LS mean improvement relative to placebo (5.92–5.96; Table 3) did not achieve the 8.5% minimally important difference (between groups) reported for Crohn’s disease [22]. Improvements from baseline were seen in the mobility and usual activities dimensions of the EQ-5D-5L (Fig. 2). The benefit of tanezumab contrasts with the poorer functional outcomes associated with persistent opioid use in patients with OA [35].

At baseline in the current study, the overall work impairment (60.41–61.07%; Table 1) of the employed subgroup was less than that reported for patients with moderate or severe OA of various joints taking prescription medication in a cross sectional study of patients in Europe (~ 79%) [3]. Work time missed over the last 7 days was also low at baseline in the current population (6.64–7.75%; Table 1) compared with the rate of absenteeism in that study (~ 59%) [3]. Differences in the version of the questionnaire used may account for some of these differences: the current study used the WPAI-OA (work time missed over the last 7 days due to OA) whereas the European study used the general health version (WPAI-GH: work time missed over the last 7 days due to “one’s health”). Improvements in work productivity (percent overall work impairment) were significantly greater for tanezumab than placebo in the current analyses, with the LS mean improvements relative to placebo (7.44–8.37; Table 3) exceeding the 7.3% minimally important difference reported for Crohn’s disease [22]. Even with the low baseline values, reductions in work time missed were significantly greater for the tanezumab 5 mg group compared with placebo, but did not reach significance for the tanezumab 2.5 mg group. Prospective intervention studies in OA using the WPAI are lacking, although imputed improvements in work productivity were reported for tapentadol compared with placebo [36].

There were few differences between the two tanezumab-treated groups in the current analyses, and the pooling strategy may be a factor in this. The similarity in design of the two studies, including eligibility criteria, assessments, and endpoints, makes the data set valuable for pooling. However, the dosing regimens differed, and data from the Study 1 dose-titration arm (tanezumab 2.5 mg at baseline and tanezumab 5 mg at week 8) were pooled with the Study 2 tanezumab 5 mg group for analyses at week 16. Potentially, the Study 1 dose-titration arm could have reduced the treatment effects seen for the pooled tanezumab 5 mg group.

The limitations of the current findings include their exploratory nature. The studies were powered for their primary endpoints, and not for these secondary endpoints. The patients recruited to the two studies differed geographically and the impact of these different healthcare systems and work cultures on the data are not known; subgroup analyses were not conducted based on geography. The employment details (jobs, industries) of the patients in the current studies were not available, precluding analyses of indirect costs.

## Conclusions

These pooled analyses showed that improvements in health status, non-work activities, and work productivity were significantly greater at week 16 following subcutaneous tanezumab administration, compared with placebo, in patients with moderate-to-severe OA.

## Supplementary Information

**Additional file 1.**

## Data Availability

Data sharing statement: Upon request, and subject to certain criteria, conditions, and exceptions (see https://www.pfizer.com/science/clinical-trials/trial-data-and-results for more information), Pfizer will provide access to individual de-identified participant data from Pfizer-sponsored global interventional clinical studies conducted for medicines, vaccines, and medical devices (1) for indications that have been approved in the US and/or EU or (2) in programs that have been terminated (ie, development for all indications has been discontinued). Pfizer will also consider requests for the protocol, data dictionary, and statistical analysis plan. Data may be requested from Pfizer trials 24 months after study completion. The de-identified participant data will be made available to researchers whose proposals meet the research criteria and other conditions, and for which an exception does not apply, via a secure portal. To gain access, data requestors must enter into a data access agreement with Pfizer.

## Abbreviations

CI: Confidence interval
KL: Kellgren-Lawrence
LS: Least squares
MDC: Minimal detectable change
NSAID: Nonsteroidal anti-inflammatory drug
OA: Osteoarthritis
PGA-OA: Patient’s global assessment of OA
WOMAC: Western Ontario and McMaster Universities Osteoarthritis Index
WPAI-GH: Work Productivity and Activity Impairment–General Health
WPAI-OA: Work Productivity and Activity Impairment–Osteoarthritis
