# Supplementary material for: Impact of tanezumab on health status, non-work activities and work productivity in adults with moderate-to-severe osteoarthritis
Source: BMC Musculoskelet Disord. 2022 Feb 1;23:106. doi: 10.1186/s12891-022-05029-x (PMC8809015; doi:10.1186/s12891-022-05029-x)
Supplement: Supplementary file 1 — Additional file 1. [file 12891_2022_5029_MOESM1_ESM.docx]

**Appendix 1- List of Institutional Review Boards**

Study 1

- Schulman Associates IRB/Advarra (main IRB, Ohio, US),
- Northwestern University IRB (Illinois, US),
- Western University Health Sciences Research Ethics Board (Ontario, Canada)

Study 2

- Ethikkommission fur das Bundesland Salzburg (Austria),
- Ethics Committee for Multicenter Trials at Ministry of Health (Bulgaria),
- Tutkimuseettinen toimikunta, Pohjois-Savon sairaanhoitopiiri (Finland),
- Comité de Protection des Personnes Sud-Est I (France),
- Geschaeftsstelle der Ethik-Kommission des Landes Berlin (Germany),
- Egeszsegugyi Tudomanyos Tanacs, Klinikai Farmakologiai Etikai Bizottsaga (Hungary),
- COMITATO ETICO INDIPENDENTE ISTITUTO CLINICO HUMANITAS (Milan, Italy),
- COMITATO ETICO REGIONALE DELLA LIGURIA (SEZIONE N. 2) (Genoa, Italy),
- Comitato Etico Interaziendale (Novara, Italy),
- COMITATO ETICO REGIONE TOSCANA - AREA VASTA CENTRO (Florence, Italy),
- Comitato Etico Indipendente (Bologna, Italy),
- COMITATO ETICO REGIONE TOSCANA-AREA VASTA SUD EST (Siena, Italy);
- Comitato Etico dell'Università “Sapienza” (Rome, Italy),
- COMITATO ETICO PER LA SPERIMENTAZIONE CLINICA DELLE PROVINCIE DI VERONA E ROVIGO (Verona, Italy),
- Medical corporation Keiaikai Saga Memorial Hospital IRB (Saga, Japan)
- Marunouchi Hospital Institutional Review Board (Nagano, Japan)
- Haradoi Hospital Institutional Review Board (Fukuoka, Japan)
- Osaka University Hospital Institutional Review Board (Osaka, Japan)
- Riverside Clinic Institutional Review Board (Hokkaido, Japan)
- Yokohama Shinmidori General Hospital Institutional Review Board (Kanagawa, Japan)
- Maebashi Hirosegawa Clinic Institutional Review Board (Gunma, Japan)
- Tokushukai Group Institutional Review Board (Tokyo, Japan)
- Kyushu Central Hospital of the Mutual Aid Association of Public School Teachers (Fukuoka, Japan)
- International Health and welfare Group - IRB, Kyushu (Fukuoka, Japan)
- National Hospital Organization CRB (Tokyo, Japan)
- Kitasato University Shirokane Institutional Review Board (Tokyo, Japan)
- Hiroshima Clinic Institutional Review Board (Hiroshima, Japan)
- Akita City Hospital Institutional Review Board (Akita, Japan)
- Fukui General Hospital Institutional Review Board (Fukui, Japan)
- Shinkokura Hospital IRB (Fukuoka, Japan)
- Jinbo Orthopedics IRB (Tokyo, Japan)
- Takeda Hospital Group Institutional Review Board (Kyoto, Japan)
- Hamamatsu Medical Center Institutional Review Board (Shizuoka, Japan)
- Hakodate Central General Hospital IRB (Hokkaido, Japan)
- Shinagawa East One Medical Clinic IRB (Tokyo, Japan)
- Urban Heights Clinic IRB (Kyoto, Japan)
- Adachikyousai Hospital Institutional Review Board (Tokyo, Japan)
- Komisja Bioetyczna przy Okręgowej Izbie Lekarskiej w Krakowie (Poland)
- Comissao de Etica para a Investigacao Clinica (CEIC) (Portugal)
- Comisia Nationala de Bioetica a Medicamentului si a Dispozitivelor Medicale (Romania)
- Eticka Komisia Bratislavskeho samospravneho kraja (Slovakia)
- CEIC - Parc de Salut Mar (Spain)
- Etikprovningsmyndigheten (Uppsala, Sweden)
- Regionala Etikprovningsnamnden i Goteborg (Goteborg, Sweden)
- NRES Committee North East - Newcastle & North Tyneside 2 (United Kingdom)
